# Serotonin, genetic variability, behaviour, and psychiatric disorders - a review

**DOI:** 10.3109/03009730903573246

**Published:** 2010-03-10

**Authors:** Niklas Nordquist, Lars Oreland

**Affiliations:** Department of Neuroscience, Section of Pharmacology, Uppsala University, UppsalaSweden

**Keywords:** Behaviour, development, genetic polymorphism, limbic system, MAOA, psychiatric disorder, serotonin, sexual dichotomy, SLC6A4

## Abstract

Brain monoamines, and serotonin in particular, have repeatedly been shown to be linked to different psychiatric conditions such as depression, anxiety, antisocial behaviour, and dependence. Many studies have implicated genetic variability in the genes encoding monoamine oxidase A (MAOA) and the serotonin transporter (5HTT) in modulating susceptibility to these conditions. Paradoxically, the risk variants of these genes have been shown, *in vitro*, to increase levels of serotonin, although many of the conditions are associated with decreased levels of serotonin. Furthermore, in adult humans, and monkeys with orthologous genetic polymorphisms, there is no observable correlation between these functional genetic variants and the amount or activity of the corresponding proteins in the brain. These seemingly contradictory data might be explained if the association between serotonin and these behavioural and psychiatric conditions were mainly a consequence of events taking place during foetal and neonatal brain development. In this review we explore, based on recent research, the hypothesis that the dual role of serotonin as a neurotransmitter and a neurotrophic factor has a significant impact on behaviour and risk for neuropsychiatric disorders through altered development of limbic neurocircuitry involved in emotional processing, and development of the serotonergic neurons, during early brain development.

## Genetic variability affecting serotonin levels

Two of the most studied molecules involved in regulating levels of serotonin in the brain are the serotonin transporter (5HTT, also known as SLC6A4), which transports serotonin from the extracellular space, and monoamine oxidase A (MAOA), the key enzyme responsible for degrading serotonin. Both genes encoding these proteins harbour genetic polymorphisms in their promoter regions that have been shown, *in vitro*, to affect their transcriptional activity (1–8). These genetic variants have been studied for their possible involvement in a number of psychiatric conditions and behavioural traits. The serotonin transporter polymorphism (5HTTLPR) and the monoamine oxidase A polymorphism (MAOA-LPR) have been implicated in conditions and behaviours such as depression, anxiety, aggression, alcoholism, autism, suicidality, and impulsiveness (9–15). The relation between these traits and the genetic variability in these genes has not always been consistent, and has displayed both interactions with environmental factors, and shown different effects in males and females.

The 5HTTLPR consists of a complex structured length polymorphism region (9,16). The allelic variants are usually referred to as *S* (short, low-expressing variant) and *L* (long, high-expressing variant). The *L*-allele has further been shown to encompass a single nucleotide polymorphism (SNP rs25531) that affects a putative transcription factor binding site and renders the *L*_G_-variant functionally equivalent to the *S*-allele (7,17). Interestingly, the rhesus macaque (*Macaca mulatta*) has been shown to contain an orthologous polymorphism in the serotonin transporter gene that has also been shown to have a functional effect on transcriptional activity (18).

The MAOA-LPR also constitutes a length polymorphism region upstream of the coding region (2). There are six allelic variants characterized in this locus, representing 2, 3, 3.5, 4, 5, or 6 copies of a repeat structure. Functional studies of their relative effect on transcriptional activity *in vitro* have unanimously shown that for the two most common variants, the 3-repeat allele displays lower transcriptional activity than the 4-repeat allele (2,3,8). The 2-repeat allele has been reported to have lower transcriptional activity than both the 3- and 4-repeat alleles (8), whereas the 3.5-repeat allele has been reported to be equal to the 4-repeat allele (2–3). The 5-repeat allele has given conflicting results (2–3), whereas the 6-repeat allele has not been functionally characterized. Most studies on this MAOA polymorphism have been focusing on the 3- and 4-repeat alleles since the other alleles are rare. Also for this polymorphism there is an orthologous equivalent in rhesus macaques with functionality shown in an *in vitro* assay (6).

Despite overwhelming amounts of data supporting a robust association between these polymorphisms and shaping of behavioural traits and vulnerability to develop psychiatric disorders, the molecular and biological events leading to the observed associations are still not fully understood. A consensus finding from association studies seems to hold that the low-expressing variants of these genes are risk factors for vulnerability to develop psychiatric disorders, conditions associated with reduced serotonergic activity, despite the fact that the low-expressing variants of 5HTTLPR and MAOA-LPR should intuitively promote increased serotonergic activity by keeping extracellular levels of serotonin high. This contradictory relation is further discussed below. Another seemingly paradoxical relation, or rather lack of relation, is based on results from *in vivo* studies on both humans and monkeys showing that the genotype in these loci does not correspond to levels of the corresponding proteins in the brain of adult individuals, as determined by positron emission tomography (PET), for either 5HTT (19–23), or MAOA (24–26), or from measurements in post-mortem brain tissues (27,28). There is a possibility, though, that an association actually exists between these polymorphisms and the abundance of the respective proteins, which might be concealed by variability in the number of expressing neurons, two measures that have not yet been simultaneously assessed. Furthermore, results from several genetic association studies suggest that these polymorphisms act differently, in some cases even in opposite directions, between the sexes (further discussed below).

## Dual functions for serotonin

Serotonin acts as a neurotransmitter released from serotonergic neurons, which emanate from cell bodies concentrated in the raphe nuclei in the brain-stem. Serotonergic projections can be traced to literally all parts of the brain. The neurocircuits making up the limbic system, involved in regulation of mood and behaviour, are particularly rich in serotonergic projections, where serotonin has been postulated to act as an inhibitor on the activity of these neuronal circuits (29). During human embryonic development, serotonin is one of the first neurotransmitters to appear, with detectable CNS neurons by 5 weeks of gestation (30). An important role for serotonin also as a trophic factor during development has been described in neural crest cells (31), heart (32), and in CNS development, which is further discussed below. Different strategies have been used to manipulate the effects of serotonin during development to understand how and where it exerts its effects. From these experiments it has become evident that regulation of serotonin homeostasis is critical for normal development of the central nervous system, where dysregulation has been linked to anatomical, functional, and behavioural anomalies.

## Serotonin excess during brain development

One of the first studies demonstrating the importance of MAOA, and consequently serotonin, in influencing human behaviour was based on an observation in a Dutch family with familial aggregation of impulsive aggression. The condition was shown to be caused by a nonsense mutation in the MAOA gene, located on the X chromosome, which caused male family members to become devoid of the enzyme (33). Both MAOA and 5HTT knock-out mouse models display elevated levels of brain serotonin during development and clearly show that this condition is associated with behavioural changes. The most prominent behavioural effect in MAOA-deficient mice is an increase in aggressiveness and impulsiveness, possibly combined with a reduction in stress response (34–36). In 5HTT knock-out mouse and rat models, the behavioural outcome is rather different from the MAOA knock-out in that it induces higher anxiety and inhibitory control, combined with reduced aggressiveness (37,38). Despite the usefulness of these models, limitations in terms of specific spatio-temporal patterns of action for the target protein have to be taken into consideration when interpreting the effects resulting from complete absence of a specific protein in a whole organism throughout all developmental stages. Using pharmaceutical inhibitors against 5HTT has been shown to cause analogous depressive-like and anxious behavioural outcomes as in 5HTT knock-out mice, treating animals either prenatally (E8–18) (39) or postnatally (P4–21) (40), which corresponds approximately to the first and the third trimester, respectively, during human foetal development (41). Inhibition of 5HTT at later time points has no observable effect on behaviour (40). Also pharmacological inhibition of MAOA during foetal development has been shown to mimic the effect seen on aggressive behaviour in MAOA knock-out mice (42). These results indicate that consequences of excessive levels of serotonin in the brain on specific behaviours later in life are, at least to some extent, determined during development.

## Serotonin deficiency during brain development

At the other end of the spectrum, clues to the involvement of serotonin in brain development and behaviour as a consequence of absence of serotonin have also been investigated. The development of serotonergic neurons is dependent on a transcriptional programme, where the ETS transcription factor Pet-1 (FEV in humans) has been shown to be a unique marker for these cells (43,44). In Pet-1-deficient mice, development of the serotonin system is abolished, rendering these animals almost completely devoid of serotonin neurons and with an 85%–90% reduction of 5HT, without evident alterations to the cytoarchitecture of the brain. Behavioural characterization of the Pet-1-deficient mice established an increased aggressive behaviour compared to wild-type control mice and also elevated anxiety-like behaviours (44). Serotonin is synthesized from the essential amino acid tryptophan by an enzyme, tryptophan hydroxylase (TPH), which is present in two forms in humans, where type 1 (TPH1) is the predominant form in peripheral tissues, whereas type 2 (TPH2) is the rate-limiting enzyme in CNS serotonin synthesis. In two recent studies, mice with either a mutant form of the Tph2 gene that caused an 80% reduction in enzyme activity, or with a disrupted gene resulting in absence of the enzyme, were characterized (45–46). These genetic modifications to the Tph2 gene caused an 80% reduction of CNS serotonin in the mutant mice (45) and total absence in the Tph2 knock-out mice (46). Also in these animal models, the behavioural consequences of serotonin depletion during development resulted in elevated aggressive and anxiety-like behaviours. From these experiments it seems clear that disruption of serotonin homeostasis during embryonic and foetal brain development has an impact on behavioural traits, which have been fundamental during evolution. The question arises as to which neuronal circuits become affected and how the elevated or reduced levels of serotonin cause these effects.

## Mechanisms of altered neuronal development by serotonin

A well characterized rodent brain region that displays anatomical alterations in response to elevated serotonin levels during development is the somatosensory cortex, which, in humans as well as rodents, has a well defined cytoarchitecture, corresponding to the afferent sensory projections from different areas of the body. Specifically the barrel fields in rodents, innervated by thalamocortical afferent neurons emanating from the whiskers, have been extensively studied due to the clear anatomical representation of barrel-like structures corresponding to individual snout whiskers in rodents (for review, see Killackey et al. (47)). Numerous studies in rodents have shown that both pharmacological and genetic alterations to serotonin levels during embryonic brain development affect the formation of barrels in the somatosensory cortex (48–51). This effect on cortex patterning results from the inability of thalamocortical afferent neurons properly to form branching arbours at their terminal ends (52). These neurons, which are mainly glutamatergic, exhibit a transient serotonergic phenotype during a period of the foetal development, in that they transiently express 5HTT, MAOA, and VMAT2, and actively take up extracellular serotonin, which is stored in vesicles and released at the nerve terminals. Detailed studies have revealed that the normal development of these neurons is dependent on their ability to capture serotonin through uptake via 5HTT, but is not dependent on the vesicular release of serotonin (50). Interestingly, this phenotype, present in 5HTT and MAOA knock-out mice, was rescued by simultaneous depletion of the serotonin receptor 5HT-1B (53,54), which has been shown to inhibit the release of glutamate at the thalamocortical nerve terminals (55). This indicates that the transient serotonin uptake in these neurons could serve to regulate the receptor activity by altering the levels of available substrate. This regulation of neuron development provides a good model to understand how altered levels of serotonin could exert its effects.

## Neuronal circuits affected by altered serotonin levels

*In vivo* functional studies of the human brain, using functional magnetic resonance imaging (fMRI), have provided important clues to which specific brain regions and functional connections between these that display serotonin-dependent activity differences (15,56–62). A recurrent finding in these studies has been that the amygdala displays both morphometric changes and activity response to behavioural tasks as a function of genetic variability in the promoter of both the MAOA and the 5HTT genes. The amygdala has an important role in emotional learning and processing, such as adequate reactions to potentially harmful situations (63). Healthy subjects with the low-functioning alleles of MAOA-LPR and 5HTTLPR have been shown to display increased amygdala activation to fearful stimuli in facial expression recognition tasks and also a reduced volume of the amygdala. In a study by Pezawas et al. it was shown that both the perigenual anterior cingulate cortex (pACC) and amygdala showed reduced volume in subjects with the low-functioning allele of the 5HTTLPR. Furthermore, it was shown that these subjects had lower ‘functional connectivity’ between these regions, implying that differences in emotional processing of negative stimuli could, to some extent, be explained by alterations in a neural circuit connecting these regions (58). These results were supported by a recent study by Pacheco et al. that investigated the effect of the 5HTTLPR on differences to a white matter fibre tract (uncinate fasciculus) anatomically connecting amygdala to regions of the prefrontal cortex (62). Here it was shown that carriers of the low-functioning 5HTTLPR had a reduced white matter connection, as determined by diffusion MRI, which supports the previously described reduced functional connectivity between these regions. Interestingly, a recent study by Jedema et al., investigating the orthologous 5HTT polymorphism in rhesus macaques, identified brain morphometric differences associated with the rh5-HTTLPR, analogous to what has been observed in humans (23). In two studies focusing on the MAOA-LPR (59,60), brain regions that showed genotypic association in response to emotion-stimulating tasks overlapped to some extent with those identified using the 5HTTLPR. In carriers of the low-functioning allele of MAOA, both the amygdala and anterior cingulate cortex displayed reduced volume compared to carriers of the high-functioning allele. However, morphometric and functional analyses also identified other regions affected by MAOA genotype and, most importantly, that the associations were conditional on sex. In males, but not in females, the low-functioning allele of MAOA was associated with increased volume of the lateral orbitofrontal cortex (59). Furthermore, in a task promoting retrieval of aversive memories, this study showed that amygdala activation in relation to MAOA genotype displayed opposite effects between males and females, where the low-functioning allele in males was associated with increased amygdala response. Also the hippocampus displayed sex-dependent activity response to this task, where male carriers of the low-functioning allele of MAOA had higher activity compared to the low-functioning allele, whereas females did not differ with respect to genotype. In a no-go flanker task, used to measure inhibitory control, the low-functioning allele of MAOA in males was associated with lowered activity in the anterior cingulate cortex, whereas in females no significant difference was observed. A study by Buckholtz et al. identified a neural circuit comprised of the amygdala, ventromedial prefrontal cortex, and anterior cingulate cortex. Male carriers of the low-functioning allele of MAOA showed an increased functional coupling between the ventromedial prefrontal cortex and the amygdala, mediated via the anterior cingulate cortex, in response to a facial expression recognition task (60). In summary, these fMRI studies clearly show that specific brain regions, previously implicated in emotion processing, and behaviour were functionally and anatomically affected by genetic variation in the genes for MAOA and 5HTT, indicating a potential effect of variable serotonin levels on these structures. The fact that levels and activity of the MAOA and 5HTT in the adult brain are poorly associated with genotype (19–26) argues for a developmental effect of the functional polymorphisms on these neurocircuits by regulating embryonic/foetal serotonin levels. This interpretation is supported by recent studies showing disturbances to the same corticolimbic structures resulting from absence of the serotonin transporter during development (64,65) and also from the identification of non-serotonergic neurons in various limbic areas, transiently expressing the serotonin transporter (66). Surprisingly, the last-mentioned study did not observe 5HTT marker expression in the amygdala during any developmental period. This result could indicate that the wealth of data associating the low-functioning variant of 5HTTLPR to hyper-reactive amygdala response and lower amygdala volume is related to an activity-dependent effect from the neural projections to and from this region during development, rather than a developmental effect of serotonin on the amygdala itself.

## Negative feedback on the serotonergic neurons by serotonin

Serotonin has been shown to modulate the outgrowth of terminals from serotonergic neurons in an auto-regulatory feedback loop both directly and indirectly (for review, see Whitaker-Azmitia (67)). The negative feedback loop seems to be dependent on the 5HT1A receptor, which is expressed early during development, both on serotonergic neurons and in target regions of the limbic circuitry. This means that excessive serotonin levels during brain development could have consequences not only for the neurocircuitry discussed above but also for the size and capacity of the serotonergic system itself. This could in part explain the paradoxical relation between genetic variants associated with increased levels of available serotonin (low-functioning alleles of MAOA and 5HTT) and the link to behavioural traits and psychiatric disorders associated with lower levels of serotonin in the brain and its metabolite 5HIAA in cerebrospinal fluid. Or, put another way, the low-functioning variants of MAOA and 5HTT could be associated with an increased risk for psychiatric disorders due to increased levels of serotonin during CNS development, causing functional alterations to neurocircuits critical for emotional processing while simultaneously inhibiting the outgrowth of the serotonergic system.

## The effect of serotonin in adulthood

Although much of the discussion above focuses on the role of serotonin during brain development and the behavioural consequences, it also has an evident role in shaping mood and behaviour in adulthood. Two well studied experimental paradigms for exploring phenotypic effects of altered serotonin levels in adulthood are ‘tryptophan depletion’ and ‘tryptophan challenge’ (68). These methods for altering central serotonin levels have specifically been used to study depression, since this condition has been strongly associated with reduced serotonergic neurotransmission. The tryptophan depletion paradigm has repeatedly been associated with a lowering of mood (68,69), assumed to be a consequence of decreased serotonin synthesis. Recent studies, however, have provided results that support a more multi-faceted function of serotonin in adulthood. A study performed on healthy women exposed to tryptophan depletion showed reduction in mood compared to controls, but this effect was shown to be moderated by genotype in the 5HTTLPR. A stress-inducing task caused a significant increase in depressive symptoms and reduced sense of vigour among carriers of the low-functioning variant of 5HTTLPR in response to tryptophan depletion, whereas the opposite effect was observed for carriers of the high-functioning variant (70). These results are in line with the observations that the low-functioning variant of 5HTTLPR has been associated with increased amygdala reactivity and reduced stress-coping (71). Since there seem to be a poor correlation between genotype at the 5HTTLPR locus and availability of the serotonin transporter in the adult brain (19–23), these differences from modulating serotonin levels in the adult brain, we hypothesize, are phenotypic consequences arising from a brain that has already been ‘wired’ by variable levels of serotonin during development. The situation seems to be even more complex considering that response to tryptophan depletion has been shown to be conditional on sex. In a study by Walderhaug et al., women reacted with a reduction in both mood and impulsiveness, whereas men showed no difference in mood but with increased impulsiveness (72). The lowering of mood among women was associated to 5HTTLPR genotype, where homozygosity for either the high- or the low-functioning variant predicted mood reduction. In this study, however, no stress-inducing task was used. The opposite paradigm, where healthy volunteers have been subjected to a tryptophan challenge, shows contradictory effects on depressive mood for females (73,74). Furthermore, also opposite genotypic effects among women were observed, where the low-functioning variant of 5HTTLPR either caused a lowering of mood (73), or an improved mood (74). In healthy men, tryptophan challenge induced a reduction in mood, which was observed to be modulated by 5HTTLPR genotype, in that carriers of the high-functioning variant scored higher on depression (73).

## Serotonin and environment interactions

Brain structures involved in emotional regulation form the neuronal foundation for how we respond to external stimuli. Above, we have outlined how serotonin could be involved in modulating some of these neurocircuits with apparent consequences for shaping of different aspects of behaviour. From this follows that genetic variability leading to altered serotonergic neurotransmission will have an impact on how individuals interpret and respond to environmental cues, such as stressful or fearful stimuli for example. However, emotional processing in this sense is not a unilateral event, determined only by the shape and activity in these neuronal circuits. In a famous study by Caspi et al., it was shown that the phenotypic outcome was modulated through an interplay between environmental insult and genetic factors (10). In a sample of male adolescents, antisocial behaviour was shown to be more common among carriers of the low-functioning variant of the MAOA-LPR, given that they had also experienced childhood maltreatment. Similar results have been observed in depression, where carriers of the low-functioning variant of 5HTTLPR were particularly vulnerable to stressful life events (11). These results imply that the penetrance of a genetic factor on behaviour seems to be dependent on the environmental context. In consecutive studies of gene–environment interactions it has been observed that the effects of variable socio-environmental factors, in combination with genotype of the MAOA-LPR and 5HTTLPR, on behavioural outcomes also display sex differences. A study by Nilsson et al. replicated the finding that males exposed to an unfavourable psycho-social environment displayed increased antisocial behaviour, particularly evident among carriers of the low-functioning variant of the MAOA-LPR (12). In a follow-up study, it was shown that, among females, this genetic variant was associated with a lower degree of antisocial behaviour (75). The findings of opposite effects from genetic variability in the MAOA-LPR between the sexes have also been revealed for alcohol-related problems in relation to poor psycho-social environment (14,76). Furthermore, a similar pattern of sexual dichotomy has been observed for the 5HTTLPR in relation to depressive symptoms (13). Interestingly, these findings have been substantiated by studies in non-human primates, showing that early rearing conditions had sex-dependent effects on stress-related response, moderated by a 5HTTLPR-orthologous polymorphism in rhesus macaques (77). Plausible explanations for the emergence of these sexually dimorphic responses might be sought among a number of different mechanisms and during various stages in development. Historically, the impact of gonadal hormones has been a prevailing model for sexually dimorphic traits, but has over time developed to include a more complex pattern of interactions between hormones, sex chromosome-linked gene expression, and environmental influences through epigenetic processes (78,79). There is a clear discrepancy in prevalence for neuropsychiatric disorders between the sexes, where, for example, females more often tend to develop major depressive disorder, generalized anxiety disorder, and post-traumatic stress disorder, whereas, in males, antisocial personality disorder, childhood attention-defecit hyperactivity disorder (ADHD), and alcohol and drug dependence are more frequent (80). Based on recent literature, an emerging consensus seems to hold that the male and female brain differ in many aspects with regard to both anatomical and functional structures (81). These differences between the sexes might indicate that the aetiology and progression in neuropsychiatric disorders could follow somewhat different paths with regard to alterations in the CNS. Furthermore, though genetic variants that cause variable levels of CNS serotonin during brain development have been shown to regulate the response to early stressful life events, these aversive stimuli, irrespectively of genetic background, also seem to affect postnatal brain development. In a study on rhesus macaques that were reared either by their mother or peers, Spinelli et al. could show that the early life stress associated with peer-rearing led to stable morphological changes in brain regions previously shown to be affected in human neuropsychiatric disorders (82). Other studies in animal models have shown that early life stress can affect neurotrophic factors such as brain-derived neurotrophic factor (BDNF) and nerve growth factor (NGF), known to be important for development of neurocircuits involved in emotional processing (83). Alterations to BDNF during postnatal brain development, in response to stress, are particularly interesting since BDNF seems to interact functionally with serotonin in brain morphogenesis (84), results which are supported by a human fMRI study where epistasis between BDNF and 5HTT was shown to affect both volume and functional connectivity within parts of the limbic circuitry (85). There are also studies in animal models showing that early life stress can affect serotonergic neurotransmission by causing expressional differences to 5HTT and serotonin receptors (86), and reducing the density of serotonin neuronal axons to specific brain regions (87).

## Concluding remarks

Serotonin has been shown to be an important factor in regulating the neuro-development of brain regions critical for emotional processing. In animal models, dysregulation of serotonin during these early neuro-developmental stages have revealed behavioural outcomes with important parallels to human neuropsychiatric disorders. Functional genetic variability in the genes for MAOA and 5HTT has been firmly associated with anatomical and functional differences in the limbic system in humans, in the absence of an association to availability or activity of the corresponding proteins, arguing for an effect of these genetic factors during brain development. Furthermore, the functional genetic variants of MAOA and 5HTT have been shown to modulate the impact of environmental factors on behavioural traits, in terms of gene–environment interactions, which in many cases display apparent sex differences. It also appears as if these interactions are reciprocal at early ages, in the sense that genetic factors regulating serotonin levels result in developmental alterations to specific neurocircuits involved in emotional processing, causing different responses to external stimuli, which has been observed to have an effect on serotonergic neurotransmission, further affecting on-going neuronal development. Future studies on the causal mechanisms behind the role of serotonin during brain development will hopefully provide knowledge, not only of how the neurocircuits involved in emotional processing work, but also give an improved understanding of the biological aetiology for neuropsychiatric disorders.
